# Antibiotic Treatment Regimes as a Driver of the Global Population Dynamics of a Major Gonorrhea Lineage

**DOI:** 10.1093/molbev/msaa282

**Published:** 2020-11-03

**Authors:** Magnus N Osnes, Lucy van Dorp, Ola B Brynildsrud, Kristian Alfsnes, Thamarai Schneiders, Kate E Templeton, Koji Yahara, Francois Balloux, Dominique A Caugant, Vegard Eldholm

**Affiliations:** 1 Division of Infectious Disease Control and Environmental Health, Norwegian Institute of Public Health, Oslo, Norway; 2 Department of Biostatistics, Institute of Basic Medical Sciences, Faculty of Medicine, University of Oslo, Oslo, Norway; 3 UCL Genetics Institute, University College London, London, United Kingdom; 4 Division of Infection Medicine, University of Edinburgh, Edinburgh, United Kingdom; 5 Department of Laboratory Medicine, Royal Infirmary of Edinburgh, NHS Lothian, Edinburgh, United Kingdom; 6 Antimicrobial Resistance Research Center, National Institute of Infectious Diseases, Tokyo, Japan

**Keywords:** *Neisseria gonorrhoeae*, antibiotic resistance, evolution, phylogeography

## Abstract

The *Neisseria gonorrhoeae* multilocus sequence type (ST) 1901 is among the lineages most commonly associated with treatment failure. Here, we analyze a global collection of ST-1901 genomes to shed light on the emergence and spread of alleles associated with reduced susceptibility to extended-spectrum cephalosporins (ESCs).

The genetic diversity of ST-1901 falls into a minor and a major clade, both of which were inferred to have originated in East Asia. The dispersal of the major clade from Asia happened in two separate waves expanding from ∼1987 and 1996, respectively. Both waves first reached North America, and from there spread to Europe and Oceania, with multiple secondary reintroductions to Asia.

The ancestor of the second wave acquired the *penA* 34.001 allele, which significantly reduces susceptibility to ESCs. Our results suggest that the acquisition of this allele granted the second wave a fitness advantage at a time when ESCs became the key drug class used to treat gonorrhea. Following its establishment globally, the lineage has served as a reservoir for the repeated emergence of clones fully resistant to the ESC ceftriaxone, an essential drug for effective treatment of gonorrhea.

We infer that the effective population sizes of both clades went into decline as treatment schemes shifted from fluoroquinolones via ESC monotherapy to dual therapy with ceftriaxone and azithromycin in Europe and the United States. Despite the inferred recent population size decline, the short evolutionary path from the *penA* 34.001 allele to alleles providing full ceftriaxone resistance is a cause of concern.

## Introduction

Gonorrhea, caused by the gram-negative bacterium *Neisseria gonorrhoeae* (the gonococcus), is a sexually transmitted disease re-emerging in large parts of the world. Globally, gonococci have acquired resistance to every drug available to treat the disease. In recent years, an increasing number of infections resistant to first-line treatments has been reported, in some instances leading to treatment failure (Unemo et al. 2012; Fifer et al. 2016). Currently, the World Health Organization recommends dual treatment with the extended-spectrum cephalosporin (ESC) ceftriaxone and the macrolide azithromycin for empirical therapy (The World Health Organization 2016). Given widespread resistance to drugs historically used to treat gonorrhea, successful treatment relies heavily on these two cornerstone drugs. So far, infections caused by strains resistant to both drugs have been sporadic and secondary transmission of these remains limited. Yet, the widespread circulation of alleles associated with reduced susceptibility to each of these drugs serves as a stern reminder that this picture could change.

The multilocus sequence type (MLST) 1901 is among the most common causes of gonococcal infections exhibiting reduced ESC susceptibility (Shimuta et al. 2015; Harris et al. 2018). Within ST-1901, the most frequent *penA* alleles associated with resistance or reduced susceptibility (RRS) to ESCs are 10.001 and 34.001 (Yahara et al. 2018). Importantly, the *penA* 34.001 allele is only a single mutation away from full ceftriaxone resistance, as demonstrated, for example, by a recent case of treatment failure in France (Unemo et al. 2012). In addition, fluoroquinolone resistance and reduced susceptibility to the second cornerstone drug, azithromycin, is widespread within the ST (Unemo et al. 2012). ST-1901 has a global distribution, but its prevalence varies between regions: it is among the most commonly observed *N. gonorrhoeae* strains in Europe, the United States, and Japan, but seems to be less frequent in Africa, with none of the current 197 African isolates in the pubMLST database (Jolley et al. 2018) belonging to the ST-1901 lineage.

The high global incidence of ST-1901 displaying reduced susceptibility to key antibiotics prompted us to perform an in-depth genomic investigation. Here, we analyze a global collection of 741 ST-1901 genomes to shed light on the historic spread of the ST, with particular focus on the emergence and spread of ESC RRS alleles. Our analyses support an East Asian origin of ST-1901, but identified North America as a central hub in the global dispersal of the strain. We also find that ceftriaxone resistant clones in Europe evolved locally from a background of reduced ESC susceptibility. Our analyses reveal consistent trends both in the routes of dispersal and in the emergence of alleles yielding reduced antibiotic susceptibility.

## Results

### Time-Resolved Phylogenetic Inferences

**Fig. 1.**
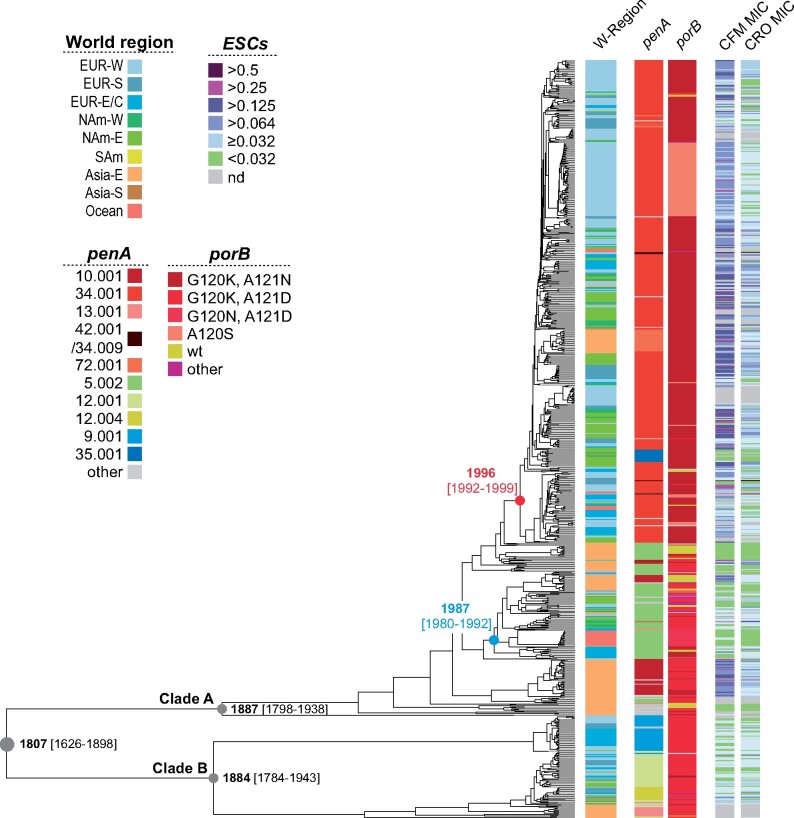
Dated genome-wide SNP phylogeny of ST-1901 annotated with region of sampling, *penA* and *porB* mutations as well as ESC MIC values for isolates where these were available. The parental nodes for two waves of expansion out of Asia are colored (Wave 1 in blue and Wave 2 in red, see text for details). CFM, cefixime; CRO, ceftriaxone; EUR-W, EUR-S, and EUR-E/C, Western, Southern, and Eastern/Central Europe, respectively; NAm-W and NAm-E, Western and Eastern North America, respectively; SAm, South America; Asia-E and Asia-S, Eastern and Southern Asia, respectively; Ocean, Oceania.

A recombination-corrected ST-1901 whole-genome phylogeny was used to generate a time-resolved phylogeny, calibrated by sample collection dates. The estimated evolutionary rate was 6.46 mutations per genome per year (CI: 5.81–7.29), translating to 2.91 × 10^−6^ (CI: 2.61 × 10^−6^ to 3.28 × 10^−6^) mutations/site/year, which falls in line with previously estimated rates for *N. gonorrhoeae* (Didelot et al. 2016; Osnes et al. 2020). A deep split at the root of the phylogeny separated ST-1901 into a major (638 out of 741 genomes) and a minor clade (fig. 1), which we termed Clade A and Clade B, respectively. These clades are inferred to have diverged sometime around the 18th century (CI: 1626–1898). The vast majority of Clade B isolates did not carry *penA* alleles associated with reduced ESC susceptibility. To assess whether the substitution rates differed between Clades A and B, we created separate dated phylogenies for the two clades, which resulted in very similar evolutionary rate estimates (6.49 [CI: 5.76–7.30] and 6.51 [CI: 4.37–9.13] mutations per genome per year for Clades A and B, respectively). Next, to investigate whether ST-1901 was truly monophyletic at the genome level, we generated a phylogenetic tree from 9,535 genomes from a global in-house database. When ST-classifications were annotated on the tree, it was clear that Clade B isolates were only distantly related to Clade A and that the overlapping MLST profiles were the result of convergent evolution and recombination (supplementary fig. S1, Supplementary Material online).

Clade A isolates were by far the more prevalent and were also associated with higher ESC minimum inhibitory concentrations (MICs). We thus chose to focus our analysis on this clade. Within the clade, East Asian isolates were typically found on deeper branches of the phylogeny compared with isolates from the rest of the world. Indeed, the majority of European and North American isolates belonged to a single recently expanded clade with a common ancestor inferred to ∼1996 (CI: 1992–1999), identifiable by the red node in figure 1.

Within Clade A, fluoroquinolone resistance was found to be universal, with 631 out of 638 isolates carrying a *gyrA* S91F in addition to either D95A or D95G and the *parC* S87R mutation. The remaining seven isolates harbored a *gyrA* S91Y mutation. The *mtrR* -35Adel promoter deletion, associated with reduced susceptibility to azithromycin and ESCs, was found in nearly all isolates of both clades, as was the *rpsJ* V57M mutation associated with tetracycline resistance (Hu et al. 2005). In addition, the *23S* 2611C>T mutation associated with azithromycin resistance, mutations in *rplD* codons 69 and 70, possibly associated with reduced susceptibility (Grad et al. 2016), and other mutations in the coding portion (G45D) and promoter region of *mtrR*, were identified in a handful of isolates across the clades (supplementary fig. S2, Supplementary Material online and extended isolate data available at http://10.6084/m9.figshare.12423596).

### Alleles Associated with Reduced ESC Susceptibility

**Fig. 2.**
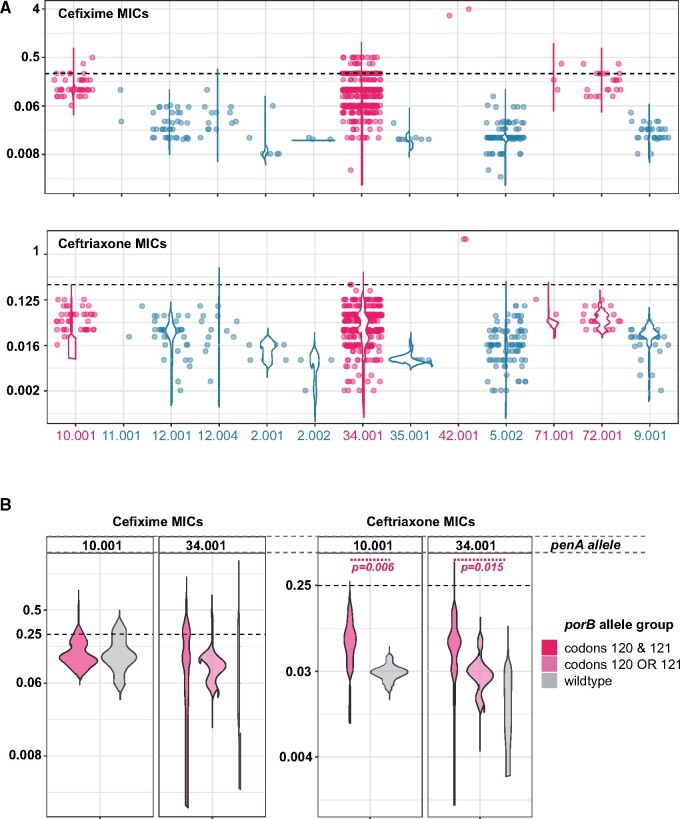
Extended-spectrum cephalosporin MICs as a function of *penA* and *porB* allele. (*A*) Observed MIC values across all *penA* alleles, only including alleles with at least two MIC measurements (609 total observations) The dotted horizontal line marks the clinical breakpoint for cefixime and ceftriaxone (0.25 mg/µl). RRS alleles are colored dark-red. (*B*) Impact of *porB* alleles on ESC MICs in RRS *penA* genetic backgrounds. For *penA* alleles 34.009, 42.001, 71.001, and 72.001, there were too few observations for statistical inference. Mann–Whitney–Wilcoxon tests were performed to assess whether MICs varied significantly by *porB* allele type.

In order to investigate the distribution and spread of alleles contributing to reduced ESC susceptibility, we identified and annotated *penA*, *ponA*, and *porB* alleles across the data set. We identified a diverse set of *penA* alleles, with a dominance of *penA* 34.001 (fig. 1). ESC MIC values were available for 609 of the 741 isolates. Based on the observed MIC measurements, we defined alleles 10.001, 34.001, 34.009, 42.001, 71.001, and 72.001 as RRS *penA* alleles (fig. 2*A*; note that the 34.009 allele was identified in a single isolate and thus excluded from the figure). In addition, we considered the allele 13.001 as an ESC RRS allele based on published findings (Liao et al. 2011; Osnes et al. 2020), despite the lack of MIC data for isolates carrying it in this study.

We found significant variation in MIC distributions within groups of isolates carrying the same *penA* allele. Isolates carrying RRS *penA* alleles typically exhibited MICs around the resistance breakpoint for the ESC cefixime (EUCAST resistance breakpoint ≥ 0.25 µg/ml for both cefixime and ceftriaxone [EUCAST 2020]). For ceftriaxone, very few isolates reached the breakpoint, with a total of four fully resistant isolates carrying 34.009 (*n* = 1) and 42.001 (*n* = 3) as notable exceptions.

In addition to the major ESC susceptibility determinant *penA*, mutations in *ponA* encoding the penicillin-binding protein 1 and the porin-encoding *porB* can modulate ESC susceptibility (Demczuk et al. 2020). In our ST-1901 collection, all but seven isolates carried the *ponA* L421P mutation (not shown, see supplementary isolate information, Supplementary Material online). In *porB*, mutations in codons 120 and 121 have been shown to confer decreased ceftriaxone susceptibility (Liao et al. 2011; Osnes et al. 2020). To investigate the effect of different *porB* alleles in ESC RRS *penA* backgrounds, we stratified isolates carrying *penA* 10.001 and 34.001 by *porB* type. We restricted the analyses to these two *penA* alleles as they were the only ESC RRS alleles found in sufficient numbers across multiple *porB* allele backgrounds in our data set. This analysis confirmed that *porB* alleles with mutations in both codons 120 and 121 were associated with higher ceftriaxone MICs (Mann–Whitney–Wilcoxon *P* < 0.05) in both *penA* backgrounds (fig. 2*B*). No significant associations were found for cefixime.

### Geographic Dispersal Patterns

**Fig. 3.**
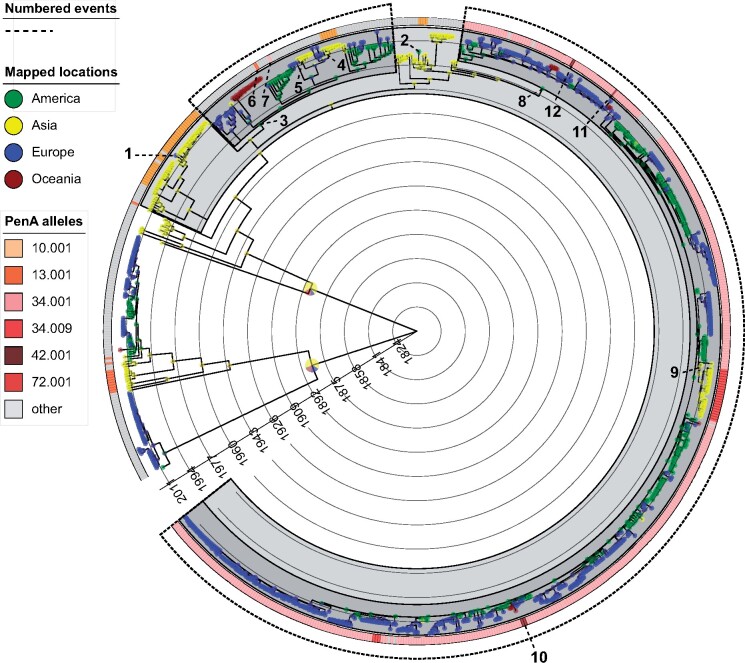
Time-resolved phylogeny of ST-1901 Clade A annotated with mapped geographical locations indicated by pie charts on nodes and tips. The outer color strip contains *penA* allele annotations. Historic events referred to in the text are indicated by stippled lines ending in event numbers.

To reconstruct the spatiotemporal dispersal history of ST-1901, the continent of isolation of each isolate was treated as a discrete trait for phylogeographic inference employing stochastic character mapping of discrete traits on phylogenies (SIMMAP) (Bollback 2006). The phylogeographic stochastic character mapping supports an East Asian origin for both Clades A and B (fig. 3), with 71.3% and 63.5% posterior probability, respectively. The uncertainty in the geographic mapping of the root (i.e., ancestor of the A and B clades) stems from the long basal branches in the tree, which allow for multiple different geographical transition histories (see supplementary fig. S3, Supplementary Material online).

For Clade A, we observed clear phylogeographic patterns pointing to the direction of global transmission events. The deeper ancestral nodes in the clade were mapped to Asia with high confidence, whereas non-Asian isolates were almost exclusively found to be descendants of two independent expansions out of Asia. Both of these waves spread to North America first, followed by subsequent exports from North America to Europe. Lineages spawned by both the first and second waves successfully established themselves in North America and Europe and have remained in endemic circulation since. Apart from these two major waves, we only observe two isolates representing independent introductions to Europe (Norway) and North America (Canada) from Asia for Clade A lineages, with no evidence of further spread (fig. 3, event number 1 and 2). Introductions to Oceania were most often seeded from Europe, but on some occasions from North America.

**Fig. 4.**
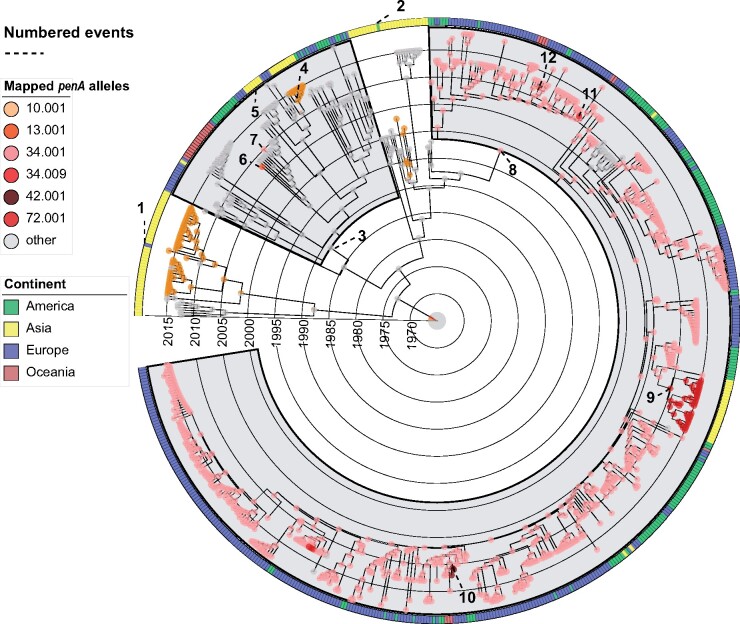
Stochastic character mapping of the different *penA* alleles on the gray subset in figure 3 leaving out Clade B and some of the basal samples from Asia in Clade A. The clades with gray background show Wave 1 and Wave 2. The color strips aligned to the phylogeny shows geographical locations (continent) of the tips. Historic events referred to in the text are indicated by stippled lines ending in event numbers.

**Fig. 5.**
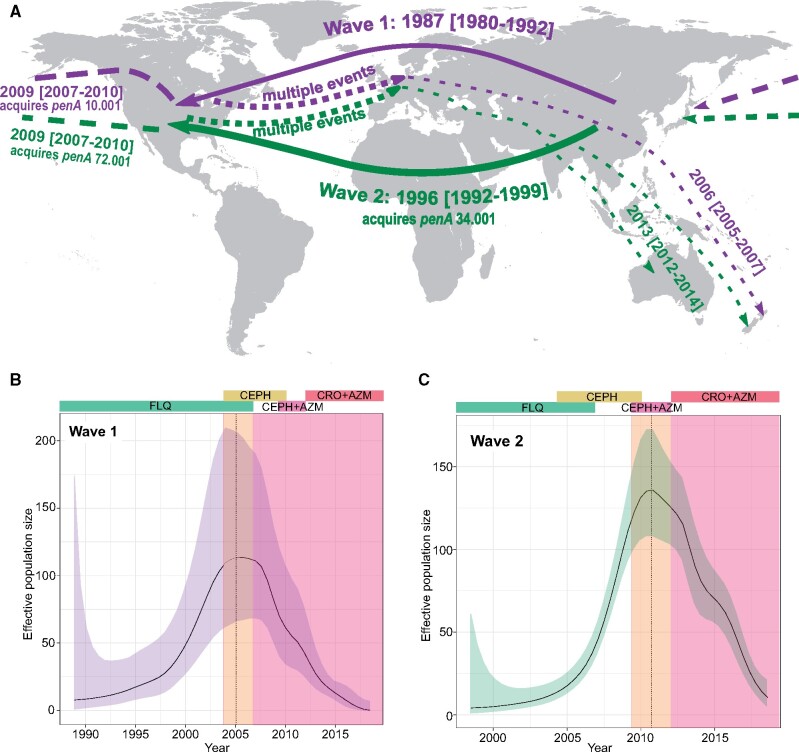
(*A*) Map showing dispersal and successful establishments of Wave 1 and Wave 2. Wave 1 emerged and spread before the acquisition of ESC RRS alleles. Wave 2 had acquired the *penA* RRS allele 34.001 prior to its global expansion. A founder event in America was important for its further and repeated introduction to Europe. (*B*) Effective population size over time estimated using Skygrowth (Volz and Didelot 2018) in the clade termed Wave 1. The vertical dotted line shows the point in time where with the maximum effective population size. The colored bars illustrate the recommended antibiotic treatments over time in the United States and Europe. FLQ, fluoroquinolones; CEPH, unspecified cephalosporin; AZM, azithromycin; CRO, ceftriaxone. The background shading in the plot illustrates the predicted effectiveness of specific antibiotics and combinations of antibiotics based on the RRS alleles present in each clade. Light orange shading indicates partial effect, darker red shading indicates strong effect. (*C*) Similar to panel B for Wave 2.

We estimated that the first wave of Clade A out of Asia originated around 1987 (CI: 1980–1992). The founder of Wave 1 (green node in fig. 1, and event number 3 in figs. 3 and 4) harbored a 5.002 allele, which was retained in almost all descendants in Europe and North America. Interestingly, two successful reintroductions from North America or Europe to Asia were inferred from this wave, both of which spawned transmission chains in Japan (figs. 3 and 4, event number 4 and 5). In one case, the strain (introduced ∼2009, fig. 3, event number 4) acquired a *penA* 10.001 allele ∼2012 (CI: 2011, 2013), associated with cefixime resistance (fig. 4, event number 4). Wave 1 also comprises lineages having independently acquired the *penA* 13.001 and 34.001 alleles in Bulgaria and the United States ∼2008 and 2010, respectively, though with no evidence of onward transmission (figs. 3 and 4, event number 6 and 7). The inferred effective population size of the Wave 1 clade was found to peak ∼2005 before starting to decline in ∼2007 (fig. 5*B*).

The second wave of Clade A ST-1901 started with a transition from Asia to North America in 1996 (CI: 1992–1999), highlighted in figure 1 with a red node and in figures 3 and 4 (event number 8). Concomitant with the expansion to North America, the lineage acquired the *penA* 34.001 allele. From this point on, the lineage rapidly spread across Europe and North America. We infer multiple acquisitions of other RRS alleles from this state, most likely driven by point mutations. Of note, the *penA* 34.001 allele is only one mutation away from either of the alleles 72.001, 34.009, and 42.001, the latter two being associated with full ceftriaxone resistance.

A reintroduction to Japan from North America around 2006 (CI: 2005–2007) is marked by the acquisition of a *penA* 72.001 allele (probably via a point mutation) and successful establishment of the lineage (figs. 3 and 4, event number 9). The median cefixime MIC for isolates harboring allele 72.001 is moderately higher than for those harboring 34.001 among our samples (0.19 vs. 0.12 µg/ml, respectively, see fig. 2), whereas the median MIC for ceftriaxone was identical between the two groups. Additional independent RRS acquisition events in this wave occurred in tandem with introductions from North America to Europe. Notable events include the acquisition of full ceftriaxone resistance via the acquisition of allele 42.001 in the second half of 2010, leading to two diagnosed cases in the United Kingdom and France (figs. 3 and 4, event number 10) and independent acquisitions of alleles 34.009 and 42.001 in Germany and Spain in patients diagnosed in 2011 and 2012, respectively (figs. 3 and 4, event number 11 and 12). We inferred that the effective population size of Wave 2 grew steadily until it peaked around 2011, following which the effective population size declined (fig. 5*B*).

To ensure that the above inferences were not overly shaped by biased sampling, we repeated the analyses on a significantly downsampled, but more geographically balanced data set (see Materials and Methods). The inferences from this data set largely recapitulated our findings above, with the exception of Wave 1, which was inferred to be the result of two export events from Asia (supplementary fig. S4, Supplementary Material online).

### Pangenomic Features of Clade A Isolates

In addition to resistance-associated genes, we investigated whether any other associated genes or alleles may have contributed to the successful expansion of Clade A, particularly the second wave. A pangenome analysis of all ST-1901 isolates, employing a 98% identity threshold identified 1,953 out of a total of 2,479 genes (79%) present in at least 95% of all analyzed genomes. Altering the identity threshold for pangenome clustering from 98% to 95% produced only marginally different summary statistics (2,471 genes in total with 1,963 present in at least 95% of the genomes); we thus considered the output from the run employing a 98% identity threshold.

Despite the recent emergence, two thirds (472 out of 741) of the ST-1901 isolates were part of the second wave of international expansion. In an effort to identify genes possibly involved in the successful expansion of Wave 2, beyond those known to be associated with drug susceptibility, the gene presence/absence matrix was scored to identify elements enriched or underrepresented in Wave 2 isolates. It was clear that the majority of identified noncore genes represented divergent alleles of the same genes rather than actual differences in gene content (supplementary table S7, Supplementary Material online). Elements differentially present in Wave 2 included both various *penA* and *porB* alleles, in addition to multiple variants of known hyper-variable genes, such as *pilE* and *piiC*, encoding a pilus assembly protein involved in adherence and an outer membrane protein, respectively. When reducing the significant hits to only those present in ≥95% of Wave 2 isolates and ≤5% of non-Wave 2 isolates or vice versa, only two genes remained, namely a *piiC* allele overrepresented in Wave 2 and a multi-species hypothetical gene (Panaroo: “group_527”), homologous to the *Neisseria meningitidis* locus NEIS0608, underrepresented in the clade.

Overall, the pangenome reconstruction revealed that there was limited variation in gene content between isolates within ST-1901. On the other hand, the circulation of highly divergent alleles, probably resulting from incorporation of DNA sequences from other *Neisseria* species (Ezewudo et al. 2015), constitutes a rich source of genetic variation, including determinants of drug susceptibility.

## Discussion

We found that the global distribution of ST-1901 Clade A, one of the most successful lineages of *N. gonorrhoeae* worldwide, was largely shaped by two waves of dispersal out of East Asia. Despite the East Asian origins of these clades, our analyses suggest that North America has played a pivotal role as a transit hub for further spread to Europe and, on occasion, back to Asia. Each of the waves was likely spawned by two singular exports out of Asia, the details of which are unknown. The successful expansions of the two clades however, were likely aided by resistance to key antibiotics.

The first wave of ST-1901 spread out of East Asia, probably in the late 1980s (1980–1992) from a background of fluoroquinolone and tetracycline resistance (mediated by chromosomal *gyrA, parC* mutations, and *rpsJ* V57M, respectively). In addition, the ancestor of Wave 1 exhibited reduced azithromycin susceptibility mediated by the *mtrR* -35Adel promoter mutation in combination with reduced susceptibility or resistance to penicillins brought about by the *ponA* L421P mutation in combination with the *penA* 5.001 allele (A517G & G543S) (Demczuk et al. 2020) (figs. 3 and 4, event number 3). In the 1980s, tetracycline and fluoroquinolones were widely used to treat gonorrhea. As a result of widespread resistance, tetracycline treatment was gradually discontinued from the late 1980s, whereas fluoroquinolones were used up until the late 1990s in many East Asian countries. In the United States, fluoroquinolone treatment was discontinued for men who have sex with men in 2004, following widespread resistance in this group, and for all patients from 2007 (Unemo and Shafer 2014).

It is thus overwhelmingly likely that the first wave of international ST-1901 dissemination was aided by resistance to fluoroquinolones. Interestingly, in one instance, the reintroduction of the strain to Japan was rapidly followed by the acquisition of a *penA* 10.001 allele around 2013 (figs. 3 and 4, event number 4), associated with cefixime resistance. This observation may suggest the presence of more favorable conditions for the evolution and transmission of cefixime resistance in East Asia compared with Europe and North America at the time. We found that the effective population size of the Wave 1 clade started declining around the time of the shift from fluoroquinolone to ESC use in the United States, consistent with new treatment regimens having played a role in hampering the spread of this clade.

The second and largest wave of global dispersal (Wave 2) started out of East Asia in the 1990s (1992–1999), with the majority of ST-1901 isolates globally descending from this event. The ancestor of Wave 2 acquired the *penA* 34.001 allele, resulting in reduced susceptibility to ESCs (figs. 3 and 4, event number 8). ESCs became the favored drugs following the shift away from fluoroquinolone use. In Japan, oral ESCs, such as cefixime, were used as monotherapy, often at relatively low concentrations (Unemo and Shafer 2014), which was probably a driver for the emergence and spread of *penA* RRS alleles. We found that the second wave of global ST-1901 dispersal was rapidly established in North America followed by multiple exports to Europe. From Europe, the strain spread further to Oceania on at least three occasions.

Similar analyses performed on a downsampled geographically balanced data set (see Materials and Methods) largely recapitulated the above findings: The credibility intervals for key node ages (supplementary table S6, Supplementary Material online) overlapped with our original inference, despite a weaker temporal signal in the downsampled data set (Root-to-tip *R*^2^ of 0.12, where higher *R*^2^ is indicative of a stronger correlation between sampling times and genetic divergence). In addition, the spatio-temporal history of Wave 2 was perfectly recapitulated, whereas the inferred first wave was split in two out-of-Asia events in the downsampled data set with independent introductions to Europe and America (supplementary fig. S4, Supplementary Material online). This split seems to be the result of the loss of informative European and American genomes. When also taking into account the stronger temporal signal of the full data set, we believe the inferences from the full data set to be more robust.

In Europe, Wave 2 representatives also evolved full-blown ceftriaxone resistance on at least three occasions (figs. 3 and 4, event number 10, 11, and 12), but these clones seem to have failed to generate significant onward transmission. The limited spread of these clones recapitulates earlier findings (Ohnishi et al. 2011) and might suggest that *penA* alleles yielding ceftriaxone resistance have a fitness disadvantage. In a pattern consistent with our observations for Wave 1, a reintroduction ∼2006 of the Wave 2 strain to Japan from North America was followed by the acquisition of a *penA* 72.001 allele from a 34.001 background (figs. 3 and 4, event number 9). Allele 72.001 is associated with further increases in cefixime MICs compared with 34.001, and the successful establishment of the strain is again indicative of stronger selection for cefixime resistance in Japan compared with Europe and North America.

In the United States, monotherapy with cefixime or ceftriaxone was widespread in the years following the discontinuation of fluoroquinolone use. In 2010, the recommended regimen was changed to dual therapy with a cephalosporin and azithromycin or doxycycline (Anon 2019). After observing both increasing cefixime MICs in the United States and cases of treatment failure abroad, the Centers for Disease Control (CDC) specified ceftriaxone as the ESC of choice in 2012 (Centers for Disease Control and Prevention [CDC] 2012). This timeline was similar in Europe, where ESC monotherapy was recommended for empirical therapy from 2009, and dual therapy mirroring the CDC recommendations from 2012 (Bignell et al. 2013). Our inferences suggest that the effective population size of Wave 2 peaked in 2011 and has since declined steeply. This suggests that the introduction of a dual treatment scheme, including ceftriaxone, succeeded in slowing the transmission of the second wave of ST-1901. In fact, the few cases of full ceftriaxone resistance we detected were inferred to have evolved in Europe during the period of ESC monotherapy (2009–2012).

Our reconstruction of the global dissemination of ST-1901 Clade A points to a central role of antibiotic-induced selection in shaping the evolution and spread of this strain, in line with other recent studies of the gonococcus (Sánchez-Busó et al. 2019; Golparian et al. 2020). We cannot rule out the existence of additional drivers of the expansion of Clade A, such as prevalence in high-risk groups, as the available metadata is lacking in this regard. In terms of genome dynamics, Clade A does not seem to stand out, as we infer nearly identical evolutionary rates and rates of recombination (supplementary fig. S5, Supplementary Material online) between Clade A and the less dispersed Clade B. Our phylogeographic analyses revealed clear, nonrandom patterns in the global movement of this ST, recapitulated by two temporally distinct waves (fig. 5). After its introduction to North America from East Asia, the second of these waves involved a large number of exports from North America to Europe. ST-1901 has spread efficiently in the Western world, and is among the most frequently observed strains in both Europe and the United States to this day. This is particularly concerning given its association with reduced ESC susceptibility (Grad et al. 2014; Harris et al. 2018).

## Conclusions

The global distribution of *N. gonorrhoeae* ST-1901 is mainly the result of two discrete waves of transmission originating in East Asia, with North America playing a central role as a transit hub for international dissemination. Strains from the largest and most recent second wave, carry a *penA* 34.001 allele which confers reduced ESC susceptibility in and of itself, but is also only a single point mutation removed from full ceftriaxone resistance. We show that resistance has evolved independently from the 34.001 background on at least three occasions in Europe, but that these events did not result in significant onward-transmission. The lack of onward-transmission of ceftriaxone resistant clones is suggestive of reduced fitness. However, compensatory mutations alleviating this fitness reduction were shown to evolve in a mouse model (Vincent et al. 2018), and it remains to be seen if similar mutants will evolve among naturally circulating gonococci. The second wave of ST-1901 dispersal led to the successful global establishment of a strain exhibiting reduced susceptibility or resistance to most drugs appropriate for gonorrhea treatment. However, we infer that the effective population size of the Wave 2 clade went into decline coinciding with the introduction of dual therapy with ceftriaxone and azithromycin in Europe and the United States. The short evolutionary path from *penA* 34.001 to full ceftriaxone resistance suggests that resistance is likely to evolve repeatedly also in the future. It will be of utmost importance to keep such clones contained.

## Materials and Methods

### Sample Collection

Genomes from the following sources were included: 1) Isolates received at the Norwegian Institute of Public Health between 2016 and 2018 that matched our typing-specific inclusion criteria (see below); 2) Isolates obtained primarily from Scotland, retained within the Scottish Bacterial STI laboratory as ST-1901 from the period 2015 to 2016; 3) Isolates from a number of published studies (Grad et al. 2014; Kwong et al. 2016; Kwong et al. 2018; Lee et al. 2018; Yahara et al. 2018; Peng et al. 2019; Sánchez-Busó et al. 2019). When typing information was not available, the genomes were screened, and those that matched our inclusion criteria retained. NCBI BioProject accessions for these are PRJNA431691, PRJDB6496, PRJNA394216, PRJNA266539, PRJEB4024, PRJEB2999, and PRJEB17738; and 4) Isolates available at PathogenWatch (https://pathogen.watch/, last accessed May 15th 2019) as of May 2019 that matched our inclusion criteria.

Only isolates with provided collection year and country of isolation were included. In addition to isolates matching the ST-1901 MLST profile, we included isolates belonging to ST-1579, ST-7360, ST-13590, and ST-8137. The first two STs have been described as single-locus variants of ST-1901 (Yahara et al. 2018) and all four STs were found embedded within the ST-1901 genome-based phylogeny in a previous analysis of Norwegian gonococcal genomes (Alfsnes et al. 2020). MLST was performed in silico (https://github.com/tseemann/mlst) using the pubMLSTdatabase (Jolley et al. 2018) to determine the ST. Extended data on all isolates are available in the Figshare repository http://10.6084/m9.figshare.12423596.

In total, the study included 139 genomes from East Asia, 414 genomes from Europe, 160 genomes from the United States, and 26 genomes from Oceania, in addition to one isolate from South America and one from the Philippines.

Ethical approval was not required as the study was initiated within the legal mandate of the Norwegian Institute of Public Health (NIPH) to investigate and report on infectious disease outbreaks. The study is restricted to the genomic analysis of microbial data and did not include patient-level data beyond country and date (year) of specimen collection.

### Genome Analyses

Raw Illumina reads were assembled de novo using SPAdes v3.13.0 (Bankevich et al. 2012) employing the “careful” mode. The assemblies were further filtered to remove contigs with a *k*-mer-coverage <3 and length <500 nucleotides. The genomes were subsequently screened with Mash v2.2.2 (Ondov et al. 2016) for the presence of contigs from other genera. Assemblies exhibiting signs of significant contamination (>100/1,000 shared hashes) with sequences from bacterial taxa other than *N. gonorrhoeae* were subsequently excluded from downstream analyses.

For comparative analyses, a high-quality reference genome (isolate 600751) was generated by hybrid assembly. Long-read data were produced on the Oxford Nanopore GridION platform as described previously (Brynildsrud et al. 2018) and short-read data were generated on the Illumina MiSeq platform. Unicycler v0.4.7 (Wick et al. 2017) was used to de novo assemble a high-quality reference genome using jointly the long Oxford Nanopore reads and short accurate Illumina reads. The resulting assembly resolved a circular genome of 2,222,926 base pairs. All short read assemblies were aligned to this closed reference genome using Parsnp v1.2 (Treangen et al. 2014). A whole-genome multifasta was generated by retaining the reference nucleotide position for all sites by filling all noncore regions with reference nucleotides using an in-house script (https://github.com/krepsen/parsnp2fasta). In total, 1,716,165 sites were retained by Parsnp, and the remaining sites filled with reference nucleotides.

Assembled genomes were annotated using Prokka v1.14.6. Prior to pangenome reconstruction, the annotated genomes were again screened with Mash (Ondov et al. 2016) as implemented through Panaroo v1.1.2 (Tonkin-Hill et al. 2020). A single genome (SRR6765327) was removed from downstream analyses on the basis of it being assembled into a high number of contigs (*n* = 397, all other assemblies were assembled into <300 contigs). In terms of genome size and gene content there was nothing suspect about the assembly, but as contig ends are generally challenging for pangenome analyses, we chose to exclude this genome. The Prokka-annotated genomes were used as input for pangenome analysis using Panaroo, employing default parameters in “strict” mode, apart from the clustering identity threshold for which two different limits were tested: 0.98 (default) and 0.95. Finally, Scoary (Brynildsrud et al. 2016) was used to identify genes with a nonrandom distribution in the clade corresponding to the second wave compared with non-Wave 2 isolates (that is, genes over or underrepresented in Wave 2 isolates). Output from the Panaroo (supplementary table S8, Supplementary Material online) and Scoary (supplementary table S7, Supplementary Material online) analyses are available as supplementary tables.

Genome clustering was performed on a large in-house global collection of 9,535 assembled genomes, using PopPUNK (Lees et al. 2019). A Neighbor-Joining tree was generated using RapidNJ as implemented in PopPUNK (Simonsen and Pedersen 2011). The phylogeny was annotated with MLST profiles generated in silico (https://github.com/tseemann/mlst).

### Temporal Phylogenetic Analyses

A maximum likelihood phylogeny was reconstructed from the whole-genome alignments using IQ-tree v1.6.9 (Nguyen et al. 2015) with 1,000 bootstrap replicates (option *-bb*). The resulting phylogeny was used as input for ClonalFrameML v1.2 (Didelot and Wilson 2015) in order to identify recombination tracts and homoplasies across the whole-genome alignments. The ratio of recombination rate to mutation rate (R/theta) was estimated at 0.42. A phylogeny annotated with identified recombination blocks is available as a supplementary figure (supplementary fig. S5, Supplementary Material online). The output from ClonalFrameML was loaded directly to BactDating v1.0.6 (Didelot et al. 2018) which accounts for branch-specific recombination rates, rather than simply ignoring recombinant regions. Root-to-tip regression with concomitant inference of the best root location (*R*^2^ = 0.17) and tip-date-randomization performed within BactDating demonstrated a clear temporal signal in the data (supplementary figs. S6 and S7, Supplementary Material online). 200 million Markov chain Monte Carlo (MCMC) steps were performed to generate a time-resolved tree using the default gamma model for clock rate. Post removal of the first 20 million steps, the resulting evolutionary rate was estimated to 6.54 substitutions/genome/year (95% CI: 5.76–7.34).

To verify the temporal inferences from the above analyses, a separate workflow was employed: Gubbins (Croucher et al. 2015) was run on the Clade A portion of the data set, followed by least squares dating using LSD2 (To et al. 2016) with 1,000 bootstrap replicates for temporal inference. The resulting TMRCA estimate for Clade A was 1,838 (CI: 1810–1861), which was well aligned with our original BactDating-inferred TMRCA in 1887 (CI: 1798–1938).

### Effective Population Size Estimates

To describe the growth and decline of the waves out of Asia we estimated their effective population size over time using the *Skygrowth* R-package v0.2.0 (Volz and Didelot 2018). *Skygrowth* uses a nonparametric Bayesian model to estimate the effective population size over time given the estimated genealogies. The model places a prior on the change in the growth rate of the effective pathogen population size, not on the change in the logarithm of the effective population size, like the commonly used *skyride* models (Minin et al. 2008). For sections of the phylogenies where data are sparse this has the advantage of using the information on the growth rate learned from the previous parts of the phylogeny, whereas the *skyride* models has a tendency to estimate stabilizing effective population sizes in such situations. We used the MCMC implementation in the R-package to fit the *Skygrowth* model. We ran one chain with 10 million steps and discarded the first 20% of the iterations as burn-in. The results were summarized using the median estimate and credibility intervals covering the 0.025–0.975 percentiles of the posterior distribution.

### Identification of Resistance Alleles

To identify mutations and alleles associated with reduced susceptibility or resistance to relevant antibiotics, whole genome assemblies were aligned against gene-specific databases downloaded from pubMLST (Jolley et al. 2018) using BLAST (Altschul et al. 1990). This was done for the following genes/segments: *penA* (NEIS1753), *ponA* (NG_ponA), *porB* (NG_porB), *mtrR* (‘mtrR – includes promoter region) *23S* (NG_23S), *parC (*NG_parC), *gyrA* (NG_gyrA), *rplD* (NEIS0133), and *rpsJ* (NEIS0129). In addition, to identify mosaic *mtrD* alleles associated with azithromycin resistance (Wadsworth et al. 2018; Williamson et al. 2019), the assemblies were blasted against the *mtrD* gene of FA1090, and the alignments visually inspected. For *penA* alleles, we used NG-STAR nomenclature (Demczuk et al. 2017).

### Phylogeography and Mutational Mapping

Geographical transition patterns over time were inferred for the time-calibrated phylogeny using stochastic character mapping (Bollback 2006) as implemented in the R-package Phytools v0.6-99 (Revell 2012). We simulated 1,000 stochastic character mappings conditional on the observed geographical states on the tips. We used four continents as categories: “North America,” “Europe,” “Asia,” and “Oceania,” to capture large distance transitions that occurred in small time intervals. The posterior probability of the geographical state of a node was calculated as the fraction of the inferred character mappings for which that node was in a given character state (continent). The times of geographical transition events were considered to be the times when the transitions happened on the branches leading to the downstream nodes with posterior probabilities >50% for new locations. Since not all simulated character histories yielded downstream nodes in the same location, the times were calculated as the average time of the simulated character histories that were compatible with the events we were interpreting (so based on at least 50% of the geographical mappings, by the criteria above). We termed a geographical transition event a “successful establishment” if it produced more than one successive downstream observation in the new location.

To describe the acquisition of the known *penA* alleles on the internal nodes of the phylogeny, we performed another stochastic character mapping analysis as previously described. Since some of the states are observed very few times in the phylogeny, leading to uncertain transition rate estimations, we defined a custom transition matrix that only estimated transition rates between groups of states with multiple observations. Noting that the rare alleles 34.009, 42.001, and 72.001 are only one point mutation away from the allele 34.001, and observing that all samples with a small genetic distance from these carried the 34.001 allele, we only allowed transitions from 34.001 to these rare alleles. The method and transition matrix estimates are described in more detail in supplementary appendix 1, Supplementary Material online. The times of transitions between allele states were calculated in the same way as for the geographical states.

### Downsampling for a Balanced Global Representation

To rule out that key spatio-temporal inferences were the result of unbalanced sampling across regions and phenotypic drug susceptibility, we generated a downsampled data set and rerun temporal and phylogeographic analyses. The downsampled data set included 90 genomes from each of the regions North America, Europe and East Asia, as well as all the genomes from Oceania (*n* = 26), the Philippines, India, and Chile (*n* = 4 in total). The European and East Asian collections were downsampled in a random fashion, whereas the North American collection was downsampled in a targeted manner to minimize the effect of targeted sampling of resistant isolates from the United States (Grad et al. 2014): We thus included all 46 phenotypically cefixime susceptible isolates in addition to 30 resistant isolates from the United States. These were complemented by 14 Canadian genomes from isolates with unknown MICs. Parsnp, Gubbins, and BactDating were employed to generate a dated phylogeny on which phylogeographic mapping was performed using SIMMAP as described above.

## Supplementary Material

Supplementary data are available at *Molecular Biology and Evolution* online.

## Supplementary Material

msaa282_Supplementary_Data
