# Corrigendum to: Antibiotic Treatment Regimes as a Driver of the Global Population Dynamics of a Major Gonorrhea Lineage

**DOI:** 10.1093/molbev/msab363

**Published:** 2022-01-13

**Authors:** Magnus N Osnes, Lucy van Dorp, Ola B Brynildsrud, Kristian Alfsnes, Thamarai Schneiders, Kate E Templeton, Koji Yahara, Francois Balloux, Dominique A Caugant, Vegard Eldholm

*Molecular Biology and Evolution*, Volume 38, Issue 4, April 2021, Pages 1249–1261, https://doi.org/10.1093/molbev/msaa282

In the originally published version of this manuscript, a second affiliation was erroneously omitted for Magnus N Osnes. His full affiliation is the following:

(1) Division of Infection Control and Environmental Health, Norwegian Institute of Public Health, Oslo, Norway

(2) Department of Biostatistics, Institute of Basic Medical Sciences, Faculty of Medicine, University of Oslo, Oslo, Norway

This error has been corrected.

