# Supplementary material for: Antibiotic Treatment Regimes as a Driver of the Global Population Dynamics of a Major Gonorrhea Lineage
Source: Mol Biol Evol. 2020 Nov 3;38(4):1249–61. doi: 10.1093/molbev/msaa282 (PMC8042733; doi:10.1093/molbev/msaa282)
Supplement: msaa282_Supplementary_Data [file msaa282_supplementary_data.zip › 1901_ Appendix 1.pdf]

Appendix 1:

Supplementary methods, tables and figures

for

"Antibiotic treatment regimes as a driver of the global population dynamics of a major gonorrhea lineage"

Table of contents

SUPPLEMENTARY METHODS AND TABLES 1

Geographical mapping 1

penA allele mapping 2-3

Comparison of dates before and after geographic downsampling 4

SUPPLEMENTARY FIGURES 5

Genome clustering of Clade A and B in a big global dataset using PopPUNK 5

Annotated phylogeny with MLST, MIC values, and mutation variants 6

Subset of the geographical stochastic character mapping 7

Geographical mapping on the downsampled dataset 8

Recombinant blocks detected using ClonalFrame 9

Root-to-tip regression 10

Date randomization tests 11

References 12

SUPPLEMENTARY METHODS

Geographical mapping

To estimate the geographical locations of the samples we used SIMMAP (1) implemented in the R-package phytools (2). We considered three transition rate models: equal rates (ER), symmetric rates (SYM), and all rates different (ARD), and selected the best transition matrix based on maximum likelihood with Akaike information criterion corrected for small sample sizes (AICc). ARD was selected as the best transition matrix having the lowest AICc of 516.8355, versus the second-best (SYM) at AICc: 556.2968 (**Table S1**). The estimated transition matrix (**Table S2**) was used to simulate 1000 stochastic character mappings and from these, the posterior node probabilities were calculated as the fraction of times the node was mapped to a certain location.

Table S1. Model comparison for geographical mapping transition matrices

Transition model	AICc	Relative likelihood	Akaike weights
All rates equal (ER)	639.5149	2.293542e-27	2.293541994e-27
Symmetric rates (SYM)	556.2968	2.698284e-09	2.698283993e-09
All rates different (ARD)	516.8355	1	9.999999973e-01

The estimated transition matrix between the geographical location is shown in **Table S2**.

Table S2. Estimated transition matrix between different geographical locations.

	America	Asia	Europe	Oceania
America	-0.128563129	0.008402323	0.11903906	0.001121746
Asia	0.005866787	-0.005866787	0	0
Europe	0.005904925	0.004924409	-0.01790133	0.007071995
Oceania	0	0.023969072	0	-0.023969072

penA allele mapping

To estimate the states of the unobserved *penA* alleles on the branches of the phylogeny we constructed a synthetic transition matrix informed by biological insight (**Table S3**). As the alleles 34.009 and 42.001 were rare, a total of 1 and 3 observations respectively, and associated with similar levels of CRO MICs, they were combined into a single common category "34.009 and 42.001". We also noted that there is only a single base pair difference between the allele 34.001 and each of the alleles 34.009, 42.001, and 72.00. In each case where 34.009, 42.001, and 72.001 appear in the phylogeny, all the closest tips have the 34.001 alleles. Therefore, we constructed the transition matrix that only allows transitions from the state 34.001 to the states 72.001 and "34.009 and 42.001", which we treated as absorbing states. We also tested if this transition matrix was supported by the data using AICc and compared it to ARD, SYM, and ER. The model had an AICc value of 315.20 comparable to the next best SYM of 315.66. All the other transition matrices considered had AICc values 2 greater than these. Since we wanted to interpret the evolutionary pattern of the alleles on the phylogeny in a way that allows the alleles to evolve with asymmetric rates, we presented the results for the synthetic transition matrix. We note that the dates of transitions and the posterior probabilities are almost identical to those obtained from the SYM model.

Table S3. The synthetic that was used to estimate the transition rates.

To<br>From	10.001	13.001	34.001	34.009 and 42.001	72.001	other
10.001	p_{11}	p_{12}	p_{13}	*0*	*0*	p_{16}
13.001	p_{21}	p_{22}	p_{23}	*0*	*0*	p_{26}
34.001	p_{31}	p_{32}	p_{33}	p_{34}	p_{35}	p_{36}
34.009 and 42.001	*0*	*0*	*0*	p_{44}	*0*	*0*
72.001	*0*	*0*	*0*	*0*	p_{55}	*0*
other	p_{61}	p_{62}	p_{63}	*0*	*0*	p_{66}

Table S4. The estimated transition rates using the transition matrix in Table S3.

	10.001	13.001	34.001	34.009 and 42.001	72.001	other
10.001	*-0.044019103*	0	0	0	0	*0.032036774*
13.001	0	-0.024427353	0	0	0	0.012445025
34.001	0	0	-0.026741643	0.003322996	0.00443229	0.007004028
34.009 and 42.001	0	0	0	-0.011982329	0	0
72.001	0	0	0	0	-0.01198233	0
other	0.002922158	0.003958928	0.001424249	0	0	-0.020287664

Table S5. Genetic SNP distance between the different penA alleles

**Allele**	10.001	13.001	34.001	34.009	42.001	72.001
10.001	0	218	13	14	14	14
13.001	218	0	205	206	206	204
34.001	13	205	0	1	1	1
34.009	14	206	1	0	2	2
42.001	14	206	1	2	0	2
72.001	14	204	1	2	2	0

Table S6. Dates for the historic events in Figure S3. Note that the credibility intervals are overlapping for all events for the analyses before and after downsampling.

<table>
<tr><th>Historic event</th><th>Downsampled Date (Credibility interval)</th><th>Original Date (Credibility interval)</th></tr>
<tr><td>Root (Event 1)</td><td>1663 (1337, 1833)</td><td>1807 (1626, 1898)</td></tr>
<tr><td>MRCA Clade A (Event 2)</td><td>1865 (1739, 1931)</td><td>1887 (1798, 1938)</td></tr>
<tr><td>MRCA Clade B (Event 3)</td><td>1818 (1662, 1914)</td><td>1884 (1784, 1943)</td></tr>
<tr><td>Wave 1 - American faction (Event 4)</td><td>1993 (1977, 1994)</td><td rowspan="2">1987 (1980, 1992)</td></tr>
<tr><td>Wave 1 - European faction (Event 5)</td><td>1986 (1976, 1994)</td></tr>
<tr><td>Wave 2 (Event 6)</td><td>2001 (1997, 2004)</td><td>1996 (1992,1999)</td></tr>
</table>

SUPPLEMENTARY FIGURES

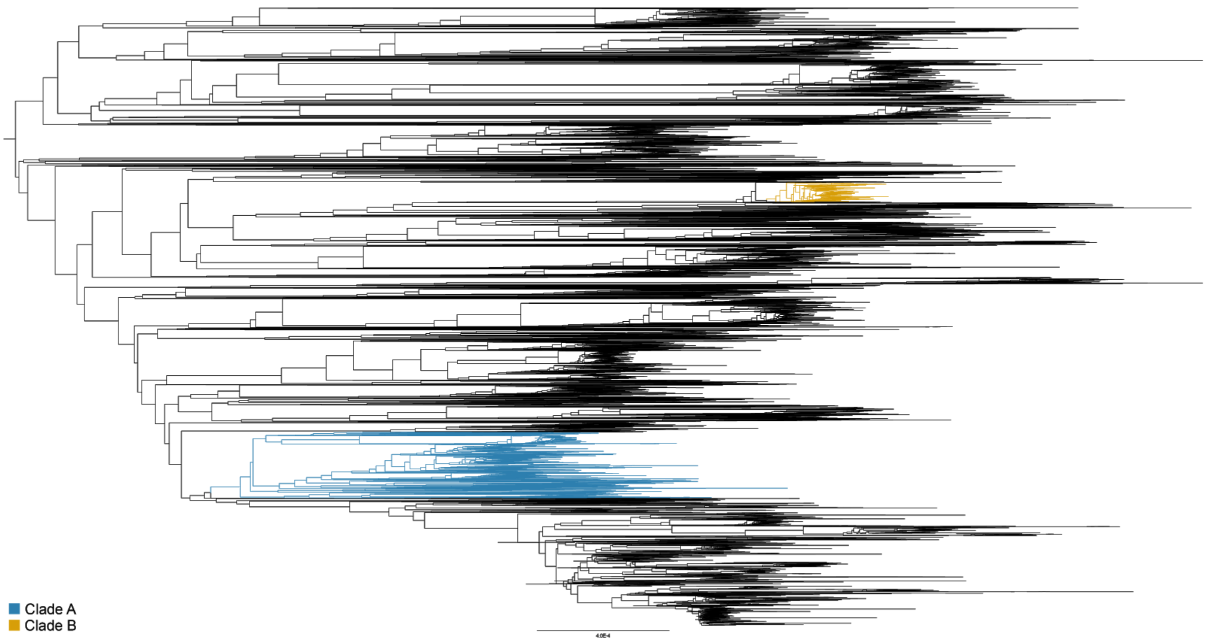

Figure S1. 9593 gonococcal genomes clustered using PopPUNK. Clades A and B are colored separately.

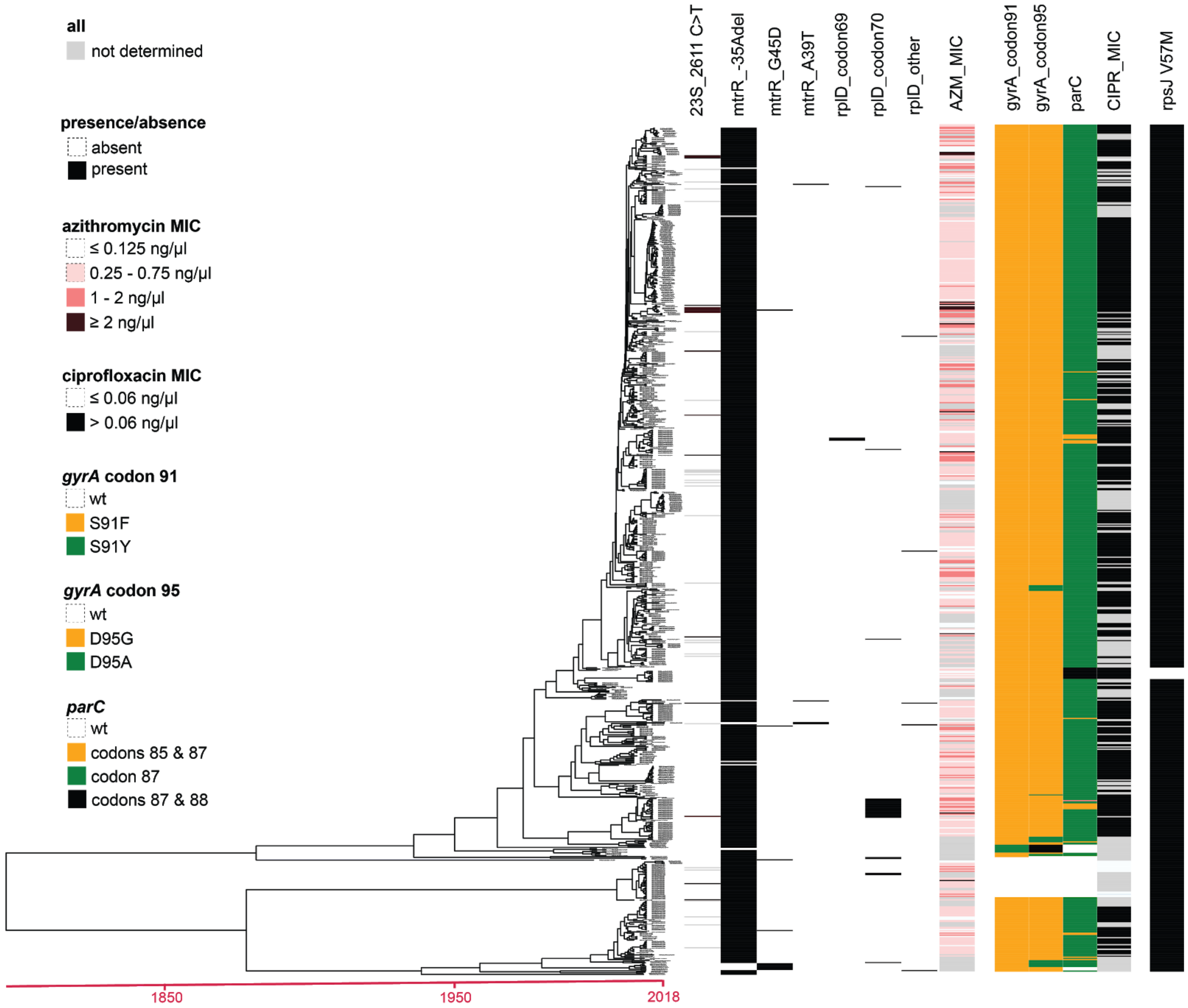

Figure S2. Dated ST-1901 phylogeny annotated with MLST profiles, mutations associated with azithromycin, ciprofloxacin, and tetracycline resistance. For azithromycin and ciprofloxacin, measured MICs are included.

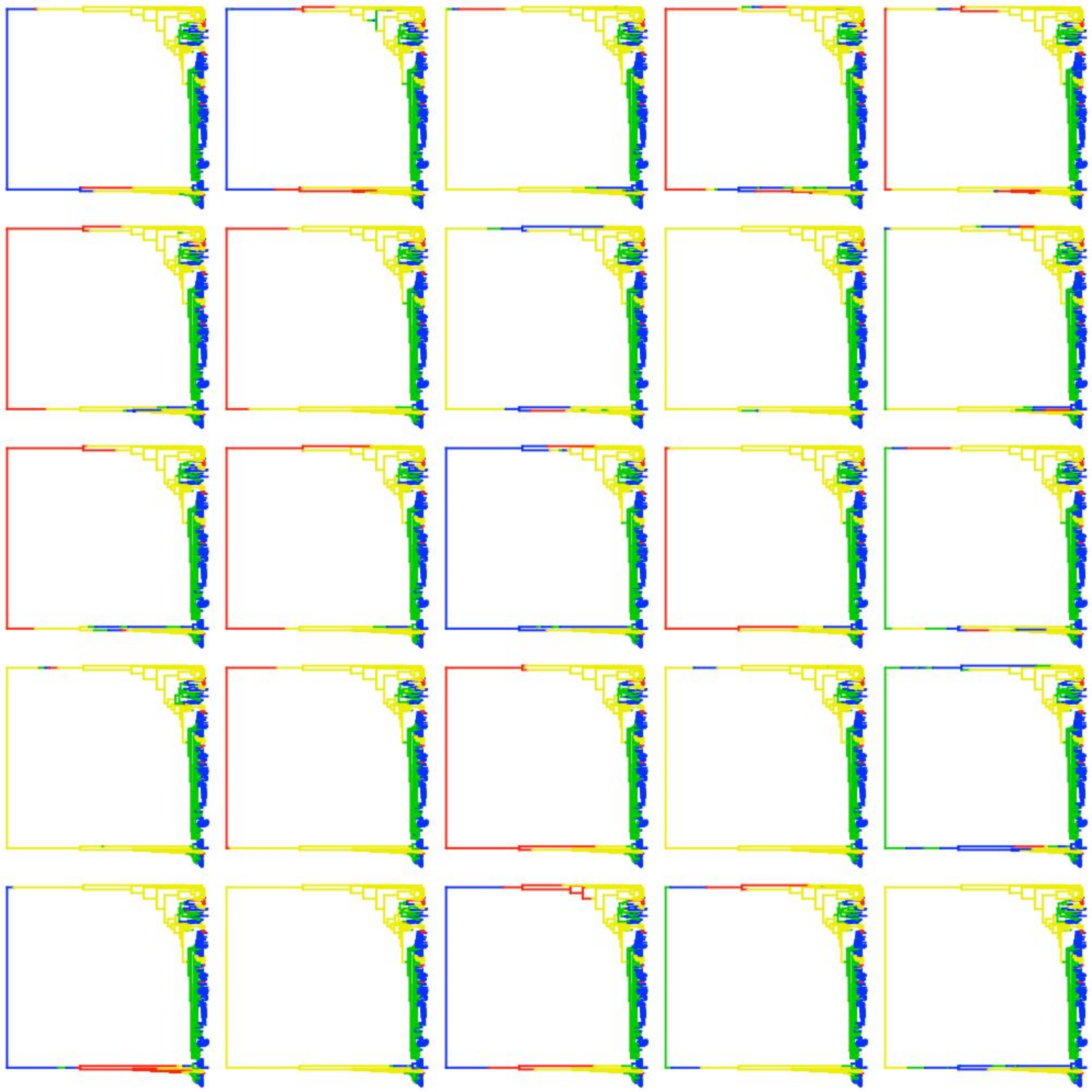

Figure S3. 25 randomly sampled stochastic character mappings out of the 1000 that were used for the phylogeographic estimation. The color indicates the continent the branches are mapped to with Asia in yellow, Oceania in red, North America in green, and Europe in blue. The phylogeny contains little information on the states on the most basal nodes, as can be seen from the highly variable color on the root node. The more recent patterns with an East Asian origin in clades A and B, and Wave 1 and Wave 2 moving out of Asia, are well preserved across the mappings.

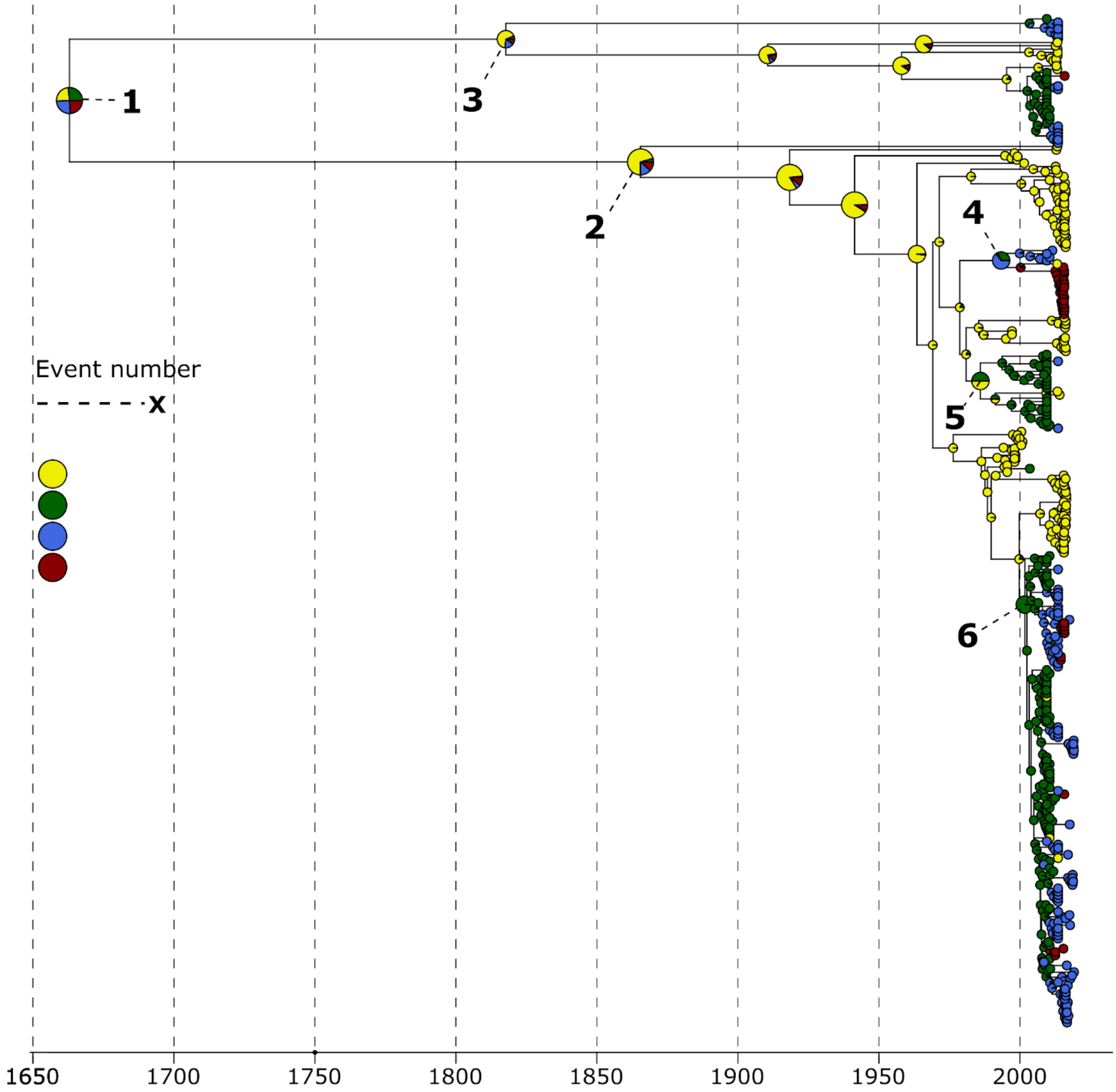

Figure S4. Phylogeographic inference on the downsampled dataset. Historic events that are discussed in the text are denoted by event numbers in the figure. The long basal branches yielded little information on the location of the root (event number 1). The most recent common ancestor (MRCA) of Clade A and B are both mapped to Asia with a high probability (event number 2 and 3). The downsampling removed some of the European and American samples in Wave 1, and this caused the wave to be split into two introductions from Asia: one to Europe (event number 4) which thereafter was introduced into Oceania, and one to America (event number 5). The ancestor of the Wave 2 lineage was mapped to America (Event number 6), which was followed by multiple introductions to Europe, in accord with our original analyses.

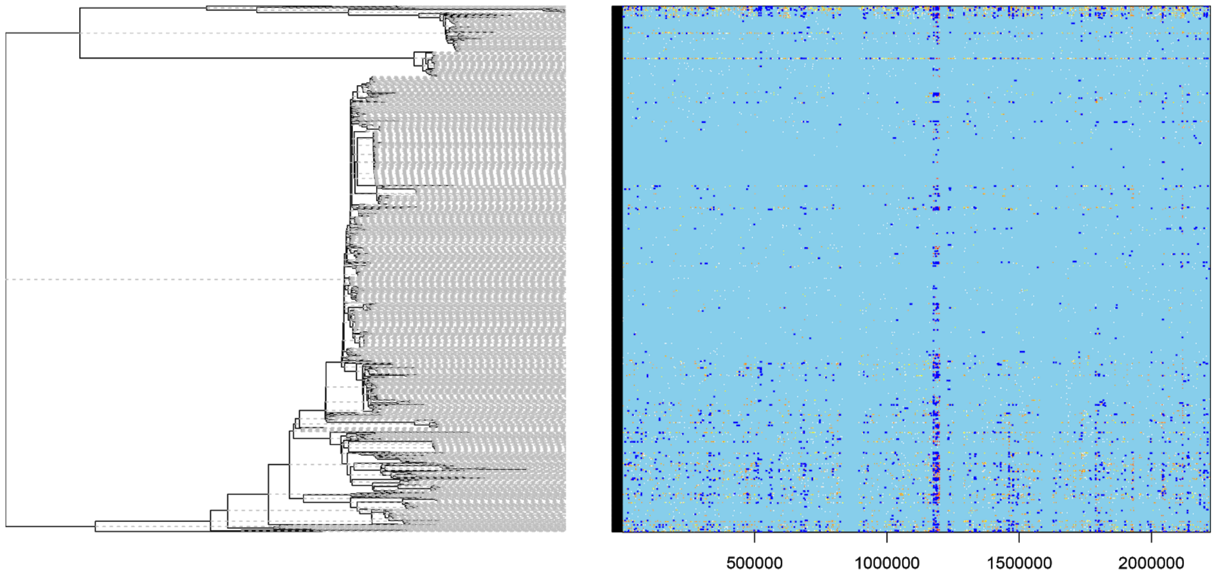

Figure S5. Recombinant blocks identified by ClonalFrameML. Recombined regions are highlighted in dark blue. Homoplasic sites are colored on a scale from yellow to red, with increasing degree of homoplasy.

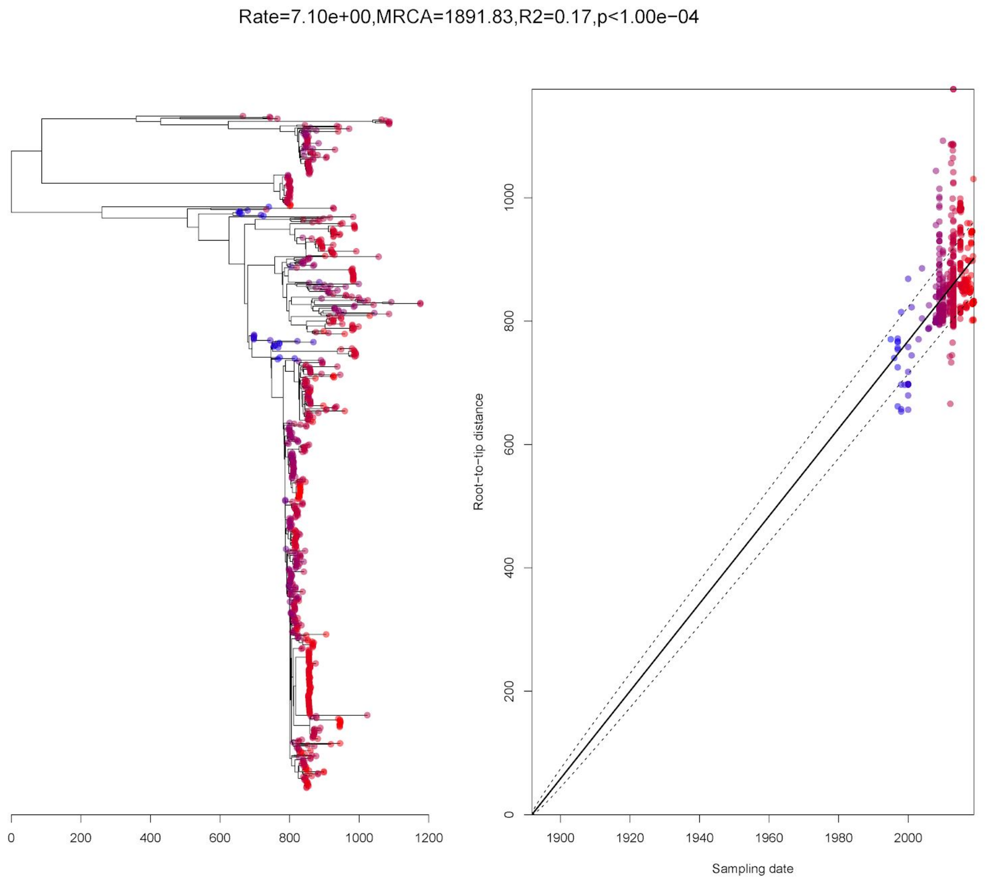

Figure S6. Root-to-tip regression performed using the *roottotip()* function provided by BactDating (3). X-axis provides the time of sampling with y providing the root-to-tip distance across the rooted maximum likelihood phylogenetic tree. BactDating provides the *p*-value following 10,000 permutations of the tree sampling dates.

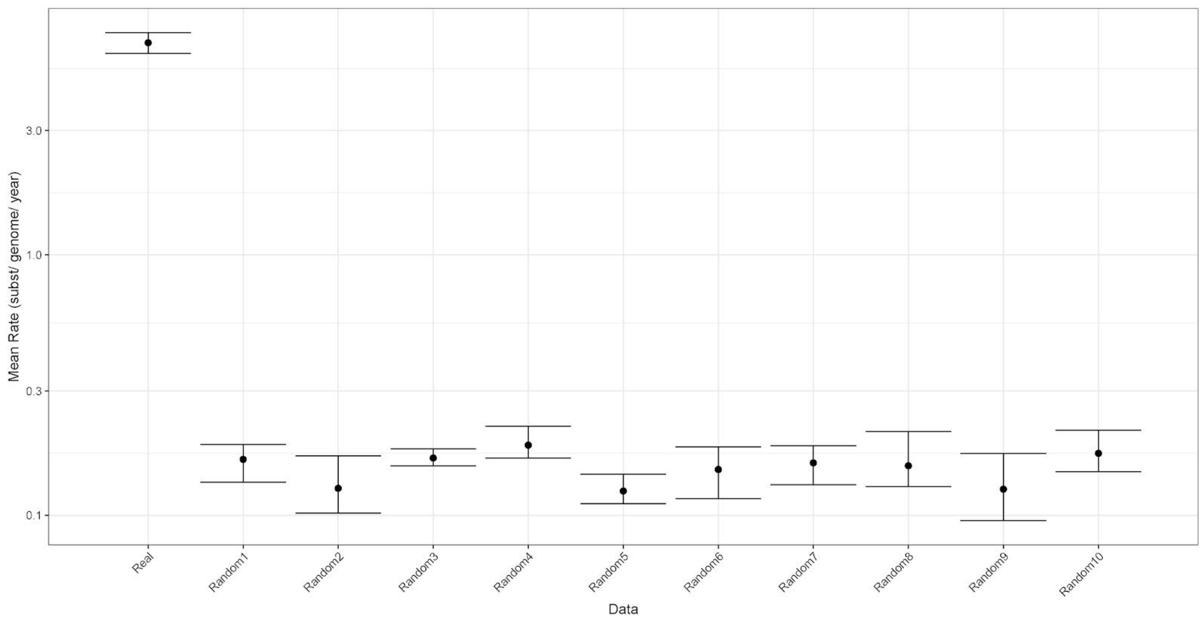

Figure S7. Evolutionary rate estimates employing real tip dates and after ten randomizations of the dates across sample tips.

References

1. Bollback JP. SIMMAP: stochastic character mapping of discrete traits on phylogenies. BMC Bioinformatics. 2006 Feb 23;7:88.

2. Revell LJ. phytools: an R package for phylogenetic comparative biology (and other things). Methods Ecol Evol. 2012;3(2):217–23.

3. Didelot X, Croucher NJ, Bentley SD, Harris SR, Wilson DJ. Bayesian inference of ancestral dates on bacterial phylogenetic trees. Nucleic Acids Res. 2018 Dec 14;46(22):e134.
